# Report of Committee on Practical Medicine

**Published:** 1851-01

**Authors:** 


### ARTICLE II.

*Report of the Committee on Practical Medicine and Epidemics.*

This report should possess a paramount interest, as designed to show the advance we are making towards the accomplishment of the ulterior objects of the profession. When a year rolls around without anything transpiring to improve our knowledge of the nature of disease, or our means of treating it, we may set it down as a period of professional barrenness. But on the other hand, when we can make the demonstration perfect of a single principle or fact bearing upon these subjects, we may console ourselves with the assurance that we are still advancing, although it may be at a very moderate pace. Tried by this standard, as exhibited in the report before us, our profession cannot be said to be making a very rapid advancement.

But we propose to inform our readers, in reference to the manner and matter of the report, and allow them to judge of the evidence of improvement for themselves.

The first three pages are principally devoted to commenting upon the Literature of Practical Medicine, and serve as an introduction to the main subject of the report—Asiatic Cholera. To this subject there are devoted twenty-two pages. The remaining four pages are occupied with references to Yellow Fever, Scarlatina, Variola, and Typhoid Fever.

There is some valuable statistical information upon the subject of Cholera in the report; especially in reference to its prevalence and treatment in Philadelphia, and her different Cholera Hospitals.

The subject of treatment is first taken up, and the success in different hands, and of different modes of practice compared.

The mortality in the seven Cholera hospitals of Philadelphia and Liberties in 1849, with a brief synopsis of treatment in each, is shown by the following summary, which we make up from the official report of each, as given in the document before us:

*Moyamensing Hospital.* The treatment at first was stimulating, and very unsuccessful, 1 in 1.54 died. After the treatment was changed and the principal reliance placed on opium in from 3 to 9 gr. doses, the mortality was only 1 in 8. These results appear to include several cases of other diseases.

*Cherry Street Hospital.* The want of success of the Calomel, opium and stimulent course of treatment, induced their abandonment. Reliance was finally placed on the free use of Quinine. Mortality of 14 in 27 cases of cholera, or 1 in 1.92 a little over one half.

*Richmond Hospital Philadelphia.* The principal reliance was on internal and external stimulents, with friction in collapsed cases, and astringents, Calomel, opium &c. in the others. Mortality, 4 in 15 cases of Cholera, or 1 in 3.75.

*Southwark Hospital.* Venesection and cups; small doses of Chloroform in camphor water, to allay nausea; dry friction for relief of spasams; opium, camphor, and sugar of lead, a a gr. j, every 1, 2 or 3 hours, to arrest alvine evacuations, were the chief remedies used here. Of 64 cholera cases, but 6 died, or 1 in 10.4.

*Northern Liberty Hospital.* Treatment not reported; morlality of cholera cases, 17 in 47, or 1 in 2.76.

*Pine St. C. Hospital.* "The treatment in this hospital was founded upon the indications as manifested by the symptoms." Mortality of cholera cases, 8 in 14, or 1 in 1,75.

*Bush Hill Hospital.* Treatment not reported. Mortality of cholera cases, 20 in 38, or 1 in 1.9.

These statistics present a very striking contrast between the Southwark Hospital, and all of the others, in the results of treatment. The contrast, too, becomes much more striking, when taken in connection with the statement of Dr. D. Francis Condie, one of the Physicians to the Southwark Hospital, that he was careful not to report a case, unless of marked character, and that his private practice was much more successful than that of the Hospital, the mortality being only four per centum.

But the summary presented in the report before us, seems to be very carelessly or unintelligibly drawn up; as, instead of corresponding to the seven Hospitals reported, there are ten ratios of mortality given. And instead of the lowest being less than ten per cent, it is put at nine, and instead of the average mortality of selected cholera cases, being 1 in 3.27, it is put down at one in 3.3.

After remarks upon different modes of practice, as pursued by different physicians, amongst the most highly recommended of which, is, the salt and mustard emetic, the subject of treatment is closed by the following judicious remarks:

"So far as the committee have been able to collect the sentiments of physicians much conversant with the subject of the treatment of cholera, they believe that the internal sedatives and astringents in moderate and frequent doses, prompt venesection, and external stimuli, including heat and cold as the case may require, and salines, constitute the most successful practice in this formidable disorder. One of the most common errors consists in the want of patience, and in the restless change of the mode of remediation, so that a medicine is not permitted to make a permanent impression, before a new article is substituted. It is chiefly for this reason that empirical remedies have so often acquired a reputation superior to that of a more rational practice which is not so steadily adhered to."

The *cause* of cholera is next discussed, and at considerable length. To fairly understand the arguments, it may be well to remember, (as the report does not divulge the fact,) that the author had previously advanced the doctrine of the cryptogamous origin of cholera, a doctrine which has for its support some plausibilities.

Passing over the arguments in reference to the modus operandi of the poison producing the disease, which assume the unity of cholera with various other diseases that occur at the same time from the unity of their causes, which is again argued from their coincidence, we shall proceed to notice the history of its introduction into this country, and as it is concise and brief, we quote it entire.

" That cholera is a *portable disease* seems now difficult to doubt. Passing over the old and oft-repeated arguments derived from the Topaz frigate, by which it seems to have been carried to the island of Mauritius, that of the Bakou, by which it appears to have been conveyed to Astracan, the Carricks, by which it seems to have reached Quebec, and the Amelia, which obviously carried it to Folly Island, we have taken some pains to collect the information necessary to understand fully the part sustained by the infected ships which arrived in the United States just at the period of the outbreak of cholera at the New York quarantine station and at New Orleans.

The infected ship which arrived first was the New York, which left Havre on the 9th of Nov., ten days after the Swanton sailed thence for New Orleans. the latter ship having weighed anchor on the 30th of Octobor. On the 25th of November, the New York was in N. Lat. 42°, Long. W. 61°, out from port 16 days, when there occurred the first case of cholera. The patient died in about 48 honrs.

One day later, 26th Nov., the Swanton, out from Port 27 days, was in latitutde 25 deg. 47 min. N. Long. 57 deg. 08 min. West, when there occcurred the first case of cholera. The patient died in about 48 hours.

The New York arrived on the 1st Dec., or in six days, and the Swanton entered the mouth of the Mississippi on the 9th, or in 13 days. On the 10th, one person died of cholera in the river, and on the 11th, just as the ship reached New Orleans, a case occurred on board, which was sent to the Charity Hospital.

The first cholera patient not attached to the ship New York was seized forty-eight hours after having been on board, and died on the same day. A nurse, who had not been on boa d and who was not known to have had any communication with the people of the New York, was also seized on the same day, and within that day died.

The first person in no way connected with the Swanton, who was attacked by cholera at New Orleans, was seized on the evening of the 13th, just forty-eight hours after the arrival of the vessel at the Levee. On the 15th, there were admitted into the Charity Hospital from the city eight cases of Cholera.

It is somewhat remarkable that, in both ships, the first cases of cholera occurred during a sudden change of the weather from an agreeable coolness to a comparatively great warmth, owing to a south wind succeeding to a northern one. The thermometer in the New York, on the 25th Nov., stood at 60°, and the Captain (Duncan) of the Swanton, in a letter to the Chairman of this Committee, says: "Previously to the commencement of the sickness, for twenty-four hours we were visited by a very hot south-east wind, such an one as I never before felt; indeed, it was more like artificially heated air than anything else." The first case came on epileptically, after which there ensued a diarrhœ, vomitings, cramps, and death.

To enable the Association the better to see the relative positions of these two vessels, the Commiitee have caused to be carefully prepared a chart, which is herewith presented. By this, it will be seen that the two vessels, so nearly simultaneously and similarly attacked, were separated by three degrees of longitude, and sixteen of latitude, or, in other words, were nearly 1000 miles asunder. Each of them retained the disease until arrival. Each sent ashore cases of cholera, and notwithstandiag the very great difference of climate and weather in Staten Island and New Orleans, the people of the respective places were attacked in the short space of forty-eight hours after arrival. It may not be unpardonable here to mention the fact that, in 1832, the Carricks brought cholera to Grosse Isle, the quarantine station of Quebec, on the 5th of June, and that on the 8th, cases began to appear in Quebec.

In whatever way these vessels may have received the infection of cholera, there is left but little doubt that they brought the cause of disease to their respective ports, and communicated it to the people on shore. There is, therefore, good reason to believe in the *portability of Cholera*."

This would seem to be pretty good argument in favor of the doctrine of the communicability of the disease, and we could see no violence either to the argument or conclusion by substituting the word *communicable* for *portable.* Nor could we see any injury from this change of terms, to the author's peculiar notions in reference to the fungeous nature of the poison, since, if fungous, it must be a specific variety of the fungi, and may be entirely dependent upon animal influences in its propogation. But the author turns immediately around and adduces arguments which he terms "numerous and cogent," against the doctrine of contagion.

But let us examine these numerous and cogent reasons. The first is found in the manner and appearance of the chol era in Philadelphia, in 1849 ; the history of which is accom panied by an outline map, showing the position of each of the first 23 cases reported to the board of health, with the date of its occurrence. By this showing it would appear that on the 30th of May, three cases were reported, and of the 17 following days, there were six days without any reports, on five days there was one case reported each day ; on one day three, and on one day four cases. It appears that they occurred in various and remote parts of the city, and liberties seeming to have little or no connection or relation one with another. Notes were addressed to the attendant physicians.

"A few of the gentlemen to whom notes were sent, failed to answer them. Two had cases which had been in the immediate vicinity of other cases, but fifteen replied decidedly that there was no reason to believe that their patients had been exposed to an infected place, or to any case or cases of cholera. One case, the eleventh, which occurred on the 12th day of the epidemic visitation, had recently visited the infected city of New York."

The facts above stated being placed in a tabular form, with the date of the occurrence of each case, the distance each was from the one that last preceded it, and the name of the attendant physician to each, compose the sum and substance of this first argument in reference to which the author observes :

A review of these facts so carefully collected and so thoroughly scrutinized, does not favor the propagation by contagion but leads rather to the conclusion that the atmosphere of the whole city had become slightly epidemical, not being sufficiently poisonous to produce its specific effects unless aided by personal imprudence, on a deleterious locality.

How carefully collected and thoroughly scrutainized in reference to the proof in hand? Are we told whether or not the disease prevailed in the city before the board of health received regular reports; as was the case in almost all other cities? Was there instituted a thorough enquiry to ascertain whether the cases had been exposed directly or indirectly to contagion? or was the non-exposure assumed as it generally has been in cities, because no one knew of exposure without the trouble of making enquiry? Are we told whether any of the cities and towns with which Philadelphia has daily and extensive intercourse in all her municipalities, were affected before the time referred to or not? No, in reference to these points, essential to the value of the statistics as bearing upon the question of contagion, the report is entirely silent. And excepting the testimony of the fifteen physicians quoted above, which for aught that appears, was given long after the cases occurred, and without any notes or particular enquiries made at the time, to aid the memory, the whole history thus paraded has very little bearing upon the point. As I have said on a former occasion, cities are not the places to investigate this question, because of the difficulty of tracing exposure.

The next evidence is a history of the disease in two families, twenty-six miles from Philadelphia, in which it committed great ravages, one after another being attacked in rapid succession. Here the possibility of contagion is not disputed, as one member of the afflicted community, with whom those first attacked had intercourse, though remaining well himself, had been to the city where cholera prevailed, about a week and one other about three weeks previously to the attack. The disease in the form of cholera did not spread beyond the two families except one case, and that had not been exposed directly, yet the neighborhood was immediately affected with diarrhœ and dysentery. The following are our author's conclusions:

"If this were a case of contagion, it must have remained latent for more than three weeks, and then have broken out simultaneously in the person who had been to Philadelphia and those who had not been there; or if assumed to have been brought by the carpet-weaver, who remained well, we are left in the difficulty of supposing that the disease is so contagious as to be carried twenty-six miles by one who does not himself suffer, and yet is so non-contagious as to fail to spread to the neighbors and visitors of persons really and sorely affected."

If it had been decided that the contagion of cholera must act in a specific time, or that it must affect those with whom it comes in contact, then these laws of its spread might furnish data for argument, and the remarks quoted would be quite pertinent; but on the abstract question of its being communicated, we confess we do not see their force.

Next we have reports of a number of cases quoted, where the cholera did not spread, though having ample opportunities. But does the fact that its spread is influenced by conditions, invalidate the evidence of its spreading by communication where it does spread?

We next have the arguments adduced against contagion in which we are surprised at an extreme looseness of expression. The first paragraph is in illustration.

"Contagious diseases, indisputably such, require a considerable period of incubation, as might be supposed where the reproduction of the poison in the system creates the disorder. No such disease is demonstratively active until after a period of at least seven, and in most cases nine days from the time of exposure."

Is not gonorrhora *demonstratively* active in less than seven days? Is it not variable in the the time of its incubation?

The second argument is that cholera is producible by imprudence in diet and filthiness. If this be true, why do we not have cholera oftener in this country than four out of the two hundred years since its settlement? Are not all manner of imprudences committed annually, even to "a heavy dinner, a prolonged debauch, or a loaded atmosphere," in all parts of our goodly land? Certainly, if the position is true we ought occasionally to meet with a case at other times than when it has been introduced into the country from abroad.

The third point is that contagious diseases more generally affect children, while cholera has a general preference for adults. The force of this position is not so apparent, though argued at considerable length. There are some contagions that do not affect children unless they are *very* forward; and some general diseases are almost wholly confined to children, one of which is croup.

These are the reasons "numerous and cogent," for believing that the cholera is not contagious, while it may be portable; which to an unsophisticated mind, seems very much like making a distinction where there is no difference.

And what do the argumhnts just stated, one, two, and three, prove except that the contagion of cholera is in some points different from that of other diseases; a fact that the most ultra contagionist could scarcely presume to deny, for although the contagion of cholera may not be governed by the same laws of other contagions, it does not in consequence follow, that it is not communicated from one to another.

There is no doubt that the poison is very active under favorable circumstances for its diffusion, and we incline to the opinion, that it may be given off in sufficient profusion to contaminate whole neighborhoods under circumstances of epidemic prevalence.

A little varied from this would seem to be the conclusion to which Dr. Condie of Philadelphia has arrived, who was formerly a very decided non-contagionist. In the April, 1850, number of the American Journal of Medical Sciences, he says:

"We nevertheless think it not improbable that, when the general predisposition to cholera already exists, the arrival of an individual laboring under the disease may be the means of its communication to the inmates of the same house or to those who have frequent intercourse with him, and that from these it may be communicated to others. But in such cases the course and spread of the disease will be very different from those in which it is produced by the general epidemic causes.

How many cases, where the cholera contagion could not be traced in cities, and where it did not extend under circumstances of exposure, would it take to disprove the positive evidence furnished by the following history, which was given me by C. W. Knott, M. D., an intelligent and veracious practitioner of the affected community. Our remote settlements and isolated communities in prairie-land, afford .the best and almost only perfect fields of observation, for determiuing the question of communicability.

Bourbonois' Grove settlement, Will Co., Ill., on the Kankakee river, contains about 2,000 inhabitants mostly French and Canadians. The settlement covers an area of about six miles square, and is completely isolated from other settlements by broad wild Prairies, from twelve to twenty miles in width in all directions, excepting on the banks of the river, which are also nearly uninhabited for nine miles above and five miles below.

This community was unusually healthy during the spring of 1849 up to the 31st of May, at which time a company of nineteen emigrants arrived from Canada. They came to Chicago in good health, tarrying two days in the city where the cholera was prevailing, and pursued their journey to their destination, losing one child by the disease on the way, (but carrying the corpse with them,) and having another child sick when they arrived. They found the neighborhood so much alarmed that no one would at first take them in, so they lodged for some time in a barn.

The next morning, June 1st, the sick child died of cholera, and one of the women was taken sick with it in the evening.

She died June 2d, in 24 hours. The night of June 3d two other children of the emigrant company were taken with it, and died in 18 hours. On the night of the 4th, a man and another woman of the same company were taken; the one died in 24 and the other in 48 hours.

Up to this time no case of the disease except it was among the members of this company had occurred in the region of country for at least a distance of twenty-five miles around, and the community there remained entirely healthy. Out of humanity Mr. Rousia took the emigrants from the barn to his house the third day after they arrived, and the last two deaths occurred at his house. The night following the death of the woman last mentioned, Mr. Rousia's little daughter was taken with the disease, and died the next day, June 7th, which was the first case among the resident population. A young woman, a connexion who nursed the little girl, was taken the day she died, and met the same fate in 24 hours. The next cases among the residents were in the family of Mr. Fabarg, a relation of some of the emigrants. They had been waiting upon the unfortunate emigrants; two of them died on the 10th, and one on the 11th of June. Mr. Allen, his wife and five children, residing in the same house, were taken with cholera within two days of the last mentioned case.

Up to this time, nearly two weeks after the arrival of the unfortunate company of emigrants, the disease was wholly confined to them and to the two houses above mentioned, notwithstanding the fearful mortality that had occurred.

Laza Jerome's daughter who was at work for Mr. Allen, was taken with the disease on the 13th, went home and her fathers family was affected with it two days afterward.

Mrs. Canran who had visited Mr. Allen's family while sick, and lived near by, was taken on the 14th, but recovered. Two of her children took it and died soon after.

Mrs. Hay who also visited Mr. Allen's family, while sick, took the disease on the 15th, and recovered. her children took it two days afterward, two of whom died.

After this time, the intercourse of the well with the sick, and the geneial prevalence of the disease were such, that its communication was traced no further.

But I cannot withhold the Doctor's account of its appearance at Rock Creek, five miles distant, the nearest settlement to that above described.

Mrs. Battachyock came from Rock Creek, her residence, to Bourbouois' Grove, to visit some relatives who were afflicted with cholera—remained over night with them, went home the next day, was taken with the disease the following night, died 36 hours afterwards. Her son and a small child were taken the next night and survived the attak but 18 hours. The next day two other of the children were taken, and lived but 12 hours. The father was attacked but recovered. A family residing half a mile distant—the man and wife being the only persons in the settlement who visited them while sick, took the disease and they lost one child.—Here there was kept up the most rigid quarantine and the disease spread no farther. Some of the neighbors went within hollowing distance and asked what they needed and placed the articles at a certain point for them to get, when they retired.

The following from Wm. Matthews, M. D., of Putnam Co., Ind., an intelligent and reliable observer, is one of a large number of such instances that have come to my knowledge, and but for want of space might be related here.

"During the prevelance of Cholera at Cincinnati, three men came up to Indianapolis on the cars, having passed through Cincinnati, and, on foot, proceeded immediately on west. Before they reached Mt. Meridan in this county, one of them had diarrhæ and vomiting, and was barely able to get to the village inn. In the course of a few hours he died, and the second also, I believe, was seized and died, the third recovering. In a few days afterward, a lady residing in the village was attacked and died; then several other cases followed and several more dying, when the disease spread to the country; and we had quite a number of cases of Cholera which apparently diverged from the transient ones brought into Mt. Meridan from the Cholera region on the Ohio."

Now, I believe, it is exceedingly proper that we have right notions about every thing; and if Cholera *is* contagious no ill consequences can possibly ensue from being made aware of the fact. In fact, with correct views concerning this point, we might, most probably, in may instances, *fence the disease in.* Small-pox we know may thus be held in check; the same doubtless might be done with Cholera, were we to entertain right notions on the subject."

But I must leave this subject.

The occurrence of Yellow Fever at Charleston, S. C., after an exemption from its ravages for ten years; and its prevalence at Rio Jainero for the first time, are the principal points noticed in reference to this disease.

The prophylactic powers of belladona in scarlantina, are especially noticed and highly spoken of.

The difficulty of understanding what is implied by Typhoid Fever, in consequence of different diseased conditions being termed Typhoid, is very properly commented upon. A definition of the term that should restrict its use to one disease, would supply a desideratum.

We do not conceive the report to be in accordance with the design of the appointment of the committee, as a large part of it is occupied in advocating one side of a professional controversy.

The sooner the committees of the Association confine themselves to the performance of the specific duties assigned them, the sooner will their reports become, what they should be, compends of the improvements in the special departments of each, as made in this country from year to year.

There is appended to the report an interesting paper by

Prof. Pancoast of Philadelphia, on the cure of certain forms of Aphonia by the inhalations of a stimulating vapor: and one from Dr. Reynolds, giving an account of the Typhoid Fever and Dysentery as they have appeared in the Epedemic form during the last four seasons on Cape Ann, but our space will not allow a further notice of them at present. E.

